# Structural Validation and Measurement Invariance of the HLS-Q12 Health Literacy Instrument in Finnish Adults: Comparing Traditional and Alignment Methods

**DOI:** 10.3389/ijph.2026.1609337

**Published:** 2026-03-26

**Authors:** Jing Zhou, Hanna Rekola, Marjorita Sormunen, Tomi Mäki-Opas

**Affiliations:** 1 School of Law, Shanghai Lixin University of Accounting and Finance, Shanghai, China; 2 Department of Social Sciences, University of Eastern Finland, Kuopio, Finland; 3 Social, Wellbeing, and Rescue Research Centre, Wellbeing Services County of North Savo, Kuopio, Finland; 4 Institute of Public Health and Clinical Nutrition, Faculty of Health Sciences, University of Eastern Finland, Kuopio, Finland

**Keywords:** alignment method, health literacy, HLS-Q12, measurement invariance, structural validation

## Abstract

**Objectives:**

To examine the internal structure, internal consistency, and measurement invariance of the HLS-Q12 across sociodemographic groups in Finnish adults, using traditional multi-group confirmatory factor analysis (MGCFA) and alignment optimization.

**Methods:**

We analyzed data from 7,077 Finnish adults drawn from a nationally representative national sample (n = 4,003) and a regional sample from North Savo (n = 3,074). Analyses included confirmatory factor analysis, MGCFA, and alignment optimization with Monte Carlo evaluation. Invariance was examined across gender, age, education, and study samples.

**Results:**

Reliability was high (α = 0.905 & ω = 0.896) and unidimensional structure (CFI = 0.951, TLI = 0.935, RMSEA = 0.058). MGCFA supported scalar invariance for gender, education, and study samples. Alignment optimization exhibited acceptable non-invariance (2.8%–25% of parameters), primarily in intercepts. Women and individuals with higher education showed higher health literacy; young adults exhibited higher levels than older cohorts.

**Conclusion:**

The Finnish HLS-Q12 supported subgroup comparisons for population monitoring, with largely adequate measurement invariance across key sociodemographic groups. The evidence pertains primarily to internal structure and measurement invariance. Further studies should examine additional validity evidence using external criteria.

## Introduction

Health literacy has increasingly been recognized as a critical determinant of health outcomes and health equity across diverse populations and systems. Defined as the knowledge, motivation, and competencies needed to access, understand, appraise, and apply health information for health-related decision-making throughout the life course [1], health literacy enables individuals to navigate complex health-related environments across society, including those with healthcare systems effectively. Recent research highlights that health literacy is not a static or purely individual trait, but a dynamic and context-dependent capability shaped by social, cultural, and organizational factors [2]. The World Health Organization [3] emphasizes that health literacy is mediated by the structures and resources of society, and that improving it requires inclusive access to education, equitable communication, and supportive environments.

Limited health literacy has been associated with a wide range of adverse health-related consequences such as poorer health outcomes and poorer use of healthcare services [4], medication errors [5], increased mortality [6], and even a higher likelihood of emergency department revisits after discharge [7].

Moreover, low health literacy disproportionately affects marginalized groups, reinforcing health disparities related to socioeconomic status, education, ethnicity, and language [5, 8]. These disparities extend across generations, as parents’ health literacy influences their children’s health-related knowledge and behaviors, creating lasting effects on family and population health [9].

The COVID-19 pandemic further highlighted the critical importance of health literacy for understanding health information and making informed health decisions [10–12]. It underscored the need for accessible, trustworthy, and culturally sensitive communication, especially in times of uncertainty and rapid change. The European Health Literacy Survey (HLS-EU) found that approximately 47% of Europeans have limited health literacy, with substantial variations between countries and population groups [13]. While the comprehensive HLS-EU-Q47 instrument advanced health literacy measurement significantly, its length posed practical challenges in surveys and clinical settings [14, 15]. To address this limitation, Finbråten et al [16] developed the HLS-Q12, a parsimonious 12-item version validated in Norwegian adults. Recent validation studies using the HLS-Q12 in Brazil [17] and rural Bangladesh [18] have confirmed cross-cultural applicability of this instrument, supporting its use in diverse contexts.

Among available health literacy instruments, we selected the HLS-Q12 for this study based on several considerations. First, the HLS-Q12 is brief and feasible for inclusion in population surveys, reducing respondent burden while retaining coverage of key health literacy domains [16]. Second, it is grounded in the European Health Literacy Survey conceptual framework, which supports cross-national comparisons [19]). Third, its focus on perceived difficulty in accessing, understanding, appraising, and applying health information aligns with our methodological aim to evaluate measurement equivalence across sociodemographic groups, which is essential for population monitoring and subgroup comparisons [20].

Despite Finland’s advanced healthcare system, universal health coverage, and high education levels, health literacy challenges persist, particularly among vulnerable groups and with evolving digital health communication demands [21, 22]. For example, older adults with multiple chronic conditions may face challenges in maintaining active engagement in health-related activities without sufficient health literacy [23]. Finnish studies have developed context-specific instruments and have applied internationally validated tools such as the HLS-EU-Q16 in selected adult samples [23], and created an instrument for school-aged children [24]. However, large-scale adult validation and the systematic use of full international instruments remain limited, constraining cross-national comparability and policy development. This represents a significant gap in Finland’s capacity to monitor population health literacy and to contribute systematically to international health literacy research.

When comparing health literacy across different demographic groups, measurement invariance becomes crucial to ensure that instruments function equivalently across populations [20, 25]. Traditional multi-group confirmatory factor analysis (MGCFA) requires strict conditions often unmet in real-world data [26]. The alignment optimization method offers a more flexible approach, accommodating partial measurement invariance while enabling meaningful group comparisons [27, 28]. This method has shown superior performance compared to traditional approaches in recent studies [29, 30].

Accordingly, we examined the internal structure, internal consistency, and measurement invariance of the HLS-Q12 in Finnish adults using MGCFA and alignment optimization. Specifically, we aimed to: (1) examine the unidimensional factor structure and internal consistency of the HLS-Q12; (2) test measurement invariance across gender, age, education, and study samples; and (3) compare findings from traditional MGCFA and alignment optimization approaches as complementary approaches for evaluating cross-group comparability.

## Methods

### Participants and Data Collection

Data were drawn from a broader health and wellbeing survey (PROM) conducted in Finland in 2024. Data collection was implemented in collaboration with two Finnish research agencies, Innolink and Bilend. A nationally representative sample (n = 4,003) was recruited via Bilend’s population panel using quota-based procedure to approximate the adult population distribution in regard to gender, age and geographical region. In addition, a complementing self-selected sample from the North Savo region was collected (n = 3,083) through multiple routes, including additional non-quota panel recruitment via Innolink and Bilend and dissemination of survey links by the Wellbeing Services County of North Savo (PSHVA) through its communication networks and outreach channels. The combined of the national and regional samples yielded N = 7,086 participants, with 7,077 cases retained after excluding participants with incomplete HLS-Q12 responses.

Participants were adults aged 18 years and older residing in Finland. All procedures were conducted in accordance with the Declaration of Helsinki and data protection regulations. The study involved anonymous online survey data collection with informed consent from all participants. Under Finnish national guidelines for ethical review in human sciences [31], formal ethics committee pre-evaluation is not required for the present study. The survey was administered online using established survey protocols with informed consent procedures.

### Measures

Health literacy was assessed using the HLS-Q12 [16] as described by Zanini et al [17]. This instrument evaluates individuals’ perceived difficulty in accessing, understanding, appraising, and applying health information across healthcare, disease prevention, and health promotion domains. Each item begins with “In your daily life, how easy or difficult is it for you to…”, followed by specific health-related tasks (e.g., “…understand information about recommended health screenings or examinations”). The instrument was translated from English into Finnish by members of the researcher team with professional proficiency in both Finnish and English and subsequently reviewed within the team to ensure clarity and conceptual consistency with the original items. Responses were rated on a 4-point Likert scale (1 = *very difficult* to 4 = *very easy)*, with higher scores indicating greater health literacy.

### Statistical Analyses

Descriptive statistics were computed using SPSS 29.0 [32]. All validation analyses were conducted using Mplus 8.11 [33] with robust maximum likelihood estimator, which provides standard errors and test statistics robust to non-normality when treating the 4-point items as approximately continuous [34]. Missing data analysis revealed a Missing at Random (MAR) pattern, addressed using Full Information Maximum Likelihood (FIML) estimation. Item-level missingness was low (0.4%–1.6%).

Psychometric validation followed a sequential approach: (1) confirmatory factor analysis (CFA) to test the unidimensional structure, (2) MGCFA to examine measurement invariance across gender, age cohorts (18–34, 35–49, 50–64, 65–74, 75–89), education levels (basic education, Vocational upper secondary education, General upper secondary education, Post-secondary non-tertiary education, college, university of applied sciences, university), and study samples (sampling source from national vs. regional), and (3) alignment optimization method as an alternative approach to handle partial measurement invariance.

Model fit was assessed using the comparative fit index (CFI), Tucker-Lewis index (TLI), root mean square error of approximation (RMSEA), and standardized root mean square residual (SRMR). Acceptable fit thresholds were CFI/TLI ≥0.90 (good fit ≥0.95), RMSEA ≤0.08 (good fit ≤0.06), and SRMR ≤0.08 (preferably ≤0.06) [35]. The chi-square-to-degrees-of-freedom ratio (χ^2^/df) was reported in Supplementary Material for completeness but not used as a primary fit indicator due to its well-documented sensitivity to large sample sizes [35, 36].

Measurement invariance was assessed using Chen’s [26] criteria: ΔCFI ≤0.01, ΔRMSEA ≤0.015, ΔSRMR ≤0.030 for metric and ≤0.010 for scalar invariance.

The alignment optimization method accommodates partial non-invariance by identifying non-invariant parameters while enabling meaningful group comparisons. Fixed alignment (ALLIGNMENT = FIXED) was implemented based on model identification requirements. Solution quality was evaluated by the proportion of non-invariant parameters, with values up to 25% regarded as favorable [27, 30]. Monte Carlo (MC) simulations (1,000 replications) validated parameter recovery, with correlation coefficient of 0.98 or greater indicating trustworthy results [27, 37].

Latent mean comparisons were conducted using a stratified approach to examine result consistency and assess potential selection bias. First, demographic group comparisons were performed using the combined sample for maximum statistical power. Subsequently, separate analyses were conducted within the national sample (n = 3,998) and regional sample (n = 3,079) to evaluate the stability of demographic patterns across sampling frames. Comparisons were limited to groups achieving adequate measurement invariance in both MGCFA (scalar level) and alignment approaches (≤25% non-invariance with acceptable Monte Carlo validation).

## Results

### Descriptive Statistics

Descriptive statistics were computed for the total sample (*N* = 7,077) and the results are exhibited in Table 1. The mean age was 49.52 years (SD = 16.27; range = 18–89), and 22.8% were aged 65 years or older. Women represented 63.6% of the sample (n = 4,472), with 36.4% men (n = 2,556). Educational attainment was diverse, 7.2% comprehensive, 30.4% vocational/technical, 9.5% high school, 11.7% college, 20.7% university of applied sciences, and 20.5% university. The national sample constituted 56.5% and the regional North Savo sample 43.5%.

**TABLE 1:** Demographic characteristics of the analytic sample (Health and wellbeing survey, Finland, 2024).

Category	Groups	Total sample	National sample	North Savo
Age (N = 7,077)	18–34	22.9	25.3	19.9
​	35–49	27.3	28.4	25.8
​	50–64	26.9	25.8	28.4
​	65–89	22.9	20.5	25.9
Gender (N = 7,028)	Male	36.4	48.1	21.3
​	Female	63.6	51.9	78.7
Education level (N = 7,073)	Basic education (comprehensive school)	7.2	8.2	5.9
​	Vocational upper secondary education	30.4	31.6	28.9
​	General upper secondary education	9.5	10.4	8.4
​	Post-secondary non-tertiary education	11.7	11.0	12.5
​	University of applied sciences	20.7	19.2	22.6
​	University	20.5	19.6	21.7
Study sample (N = 7,077)	National sample	56.5	—	—
​	Regional sample	43.5	—	—

Item-level descriptives are presented in Supplementary Table S1. Item means ranged from 2.86 to 3.53 (scale 1–4), with standard deviations (SD) between 0.59 and 0.78. Missingness was minimal (0.4%–1.6% per item), suggesting limited item nonresponse. The distribution of the total HLS-Q12 score was approximately normal with slight negative skew.

### Confirmatory Factor Analysis

An exploratory factor analysis using principal axis factoring supported a unidimensional structure for the HLS-Q12 (KMO = 0.942; Bartlett’s χ^2^(66) = 35,155.17, *p* < 0.001). A single factor with an eigenvalue greater than 1 explained 44.7% of the variance.

CFA was conducted to test the unidimensional structure. Initial model fit was acceptable (CFI = 0.923, TLI = 0.906, RMSEA = 0.068, SRMR = 0.038). Following modification indices and theoretical considerations [38], four residual covariances were added between conceptually related items, resulting in improved fit (CFI = 0.951, TLI = 0.935, RMSEA = 0.058, SRMR = 0.032).

All items loaded significantly on the latent factor, with standardized loadings ranging from 0.592 to 0.753. Detailed loadings are reported in Table 2. Internal consistency reliability was excellent (Cronbach’s α = 0.905, McDonald’s ω = 0.896), indicating strong scale reliability for measuring health literacy as a unified construct.

**TABLE 2:** Standardized factor loadings for the HLS-Q12 Items from the confirmatory factor analysis Model (Health and wellbeing survey, Finland, 2024).

Item	Standardized factor loadings	Standard error	P-value
Q1	0.673	0.009	0.000
Q2	0.682	0.008	0.000
Q3	0.701	0.008	0.000
Q4	0.592	0.009	0.000
Q5	0.685	0.008	0.000
Q6	0.618	0.010	0.000
Q7	0.678	0.008	0.000
Q8	0.674	0.009	0.000
Q9	0.712	0.007	0.000
Q10	0.656	0.008	0.000
Q11	0.753	0.007	0.000
Q12	0.621	0.009	0.000

Standardized factor loadings (λ) are reported from the final CFA, model. All loadings were statistically significant at *p* < .001. Four residual covariances were added between Q6–Q4, Q7–Q8, Q9–Q11, and Q10–Q11 to improve model fit. N = 7,077.

### Invariance Testing via Multi-Group Confirmatory Factor Analysis

MGCFA results demonstrated stable measurement properties across demographic groups. Full scalar invariance was achieved for gender, education, and sampling source with minimal fit deterioration (ΔCFI ≤0.009, ΔRMSEA ≤0.003, see Table 3). For age cohorts, the change in CFI at the scalar level was larger (ΔCFI = −0.018), whereas changes in RMSEA and SRMR were small. Taken together, these results suggest some degree of age-related non-invariance, which was further examined using the alignment approach.

**TABLE 3:** Measurement invariance of HLS-Q12 across the different groups (gender, education, age, study sample) (Health and wellbeing survey, Finland, 2024).

Group/model	χ^2^ (df)	CFI	TLI	RMSEA	SRMR	Δ*χ* ^2^ (Δdf)	ΔCFI	ΔRMSEA	ΔSRMR
Gender									
Configural model	1,318.159 (100)	0.949	0.933	0.059	0.033	​	​	​	​
Metric model	1,366.007 (111)	0.947	0.937	0.057	0.035	47.848 (11)	−0.002	−0.002	0.002
Scalar model	1,613.026 (122)	0.938	0.932	0.059	0.039	247.019 (11)	−0.009	0.002	0.004
Education level									
Configural model	1,507.401 (300)	0.950	0.934	0.058	0.035	​	​	​	​
Metric model	1,636.811 (355)	0.947	0.941	0.055	0.045	129.410 (55)	−0.003	−0.003	0.010
Scalar model	1869.337 (410)	0.940	0.942	0.055	0.050	232.526 (55)	−0.007	0.000	0.005
Age cohort									
Configural model	1,402.348 (200)	0.952	0.937	0.061	0.033	​	​	​	​
Metric model	1,526.251 (233)	0.949	0.942	0.056	0.046	123.903 (33)	−0.003	−0.005	0.013
Scalar model	2018.906 (266)	0.931	0.931	0.061	0.058	492.655 (33)	−0.018	0.005	0.012
Study sample									
Configural model	1,332.421 (100)	0.950	0.934	0.059	0.033	​	​	​	​
Metric model	1,384.450 (111)	0.948	0.938	0.057	0.036	52.029 (11)	−0.002	−0.002	0.003
Scalar model	1,502.991 (122)	0.944	0.939	0.057	0.040	118.541 (11)	−0.004	0.000	0.004

χ^2^, chi-square test statistic; df, degrees of freedom; p, probability value; CFI, comparative fit index; RMSEA, root mean square error of approximation; SRMR, standardized root mean square residual; Δχ^2^, likelihood ratio test (difference in chi-square between nested models); ΔCFI, change in CFI; ΔRMSEA, change in RMSEA; ΔSRMR, change in SRMR., Measurement invariance was evaluated following Chen’s (2007) guidelines: ΔCFI ≤0.010, ΔRMSEA ≤0.015, and ΔSRMR ≤0.030 for metric and ≤0.010 for scalar invariance. All p-value <0.001.

### Invariance Testing via Alignment Optimization Approach

The alignment analysis provided complementary insights into measurement invariance patterns (see Table 4 for full sample and Supplementary Material 2, 3 for separate sample). Education comparisons showed the highest invariance quality (2.8% non-invariant parameters), followed by national/regional sample comparisons (16.7%), age cohorts (18.8%), and gender (25.0%). Non-invariance was primarily concentrated in item intercepts rather than factor loadings, indicating consistent factor structure across groups while allowing for baseline response differences.

**TABLE 4:** Approximate measurement invariance for item intercepts and factor loadings across groups using alignment optimization (Health and wellbeing survey, Finland, 2024).

Item number	Gender		Education level						Age cohort				Study sample	
Loading invariance														
Q1	1	2	1	2	3	4	5	6	1	2	3	4	1	2
Q2	1	2	1	2	3	4	5	6	1	2	3	4	1	2
Q3	1	2	1	(2)	3	4	5	6	1	2	3	4	1	2
Q4	1	2	1	2	3	4	5	6	1	2	3	4	1	2
Q5	1	2	1	2	3	4	5	6	1	2	3	4	1	2
Q6	1	2	1	2	3	4	5	6	1	2	(3)	(4)	1	2
Q7	1	2	1	2	3	4	5	6	1	2	3	4	1	2
Q8	1	2	1	2	3	4	5	6	1	2	3	4	1	2
Q9	1	2	1	2	3	4	5	6	1	2	3	4	1	2
Q10	1	2	1	2	3	4	5	6	1	2	3	4	1	2
Q11	1	2	1	2	3	4	5	6	1	2	3	4	1	2
Q12	1	2	1	2	3	4	5	6	1	2	3	4	1	2
Loadings noninvariance %	0.0%		1.4%						4.2%				0.0%	
Intercepts invariance														
Q1	1	2	1	2	3	4	5	6	1	2	3	(4)	(1)	(2)
Q2	1	2	1	2	3	4	5	(6)	1	2	3	(4)	1	1
Q3	(1)	(2)	1	2	3	4	5	6	1	2	(3)	(4)	(1)	(2)
Q4	1	2	1	2	3	4	5	6	(1)	2	3	(4)	1	1
Q5	1	2	1	2	3	4	5	6	1	2	3	4	1	1
Q6	(1)	(2)	1	2	3	4	5	6	(1)	(2)	(3)	(4)	(1)	(2)
Q7	1	2	1	2	3	4	5	6	(1)	2	3	(4)	1	1
Q8	(1)	(2)	(1)	(2)	3	4	5	6	1	2	3	4	1	1
Q9	(1)	(2)	1	2	3	4	5	6	1	2	(3)	(4)	1	1
Q10	(1)	(2)	1	2	3	4	5	6	1	2	(3)	4	1	1
Q11	1	2	1	2	3	4	5	6	1	2	3	4	1	1
Q12	(1)	(2)	1	2	3	4	5	6	1	2	3	(4)	(1)	(2)
Intercepts noninvariance %	50.0%		4.2%						33.3%				33.3%	
Total noninvariance %	25.0%		2.8%						18.8%				16.7%	

Numbers represent group classifications: Gender (1 = male, 2 = female); Education (1 = Comprehensive school, 2 = Vocational/technical school, 3 = high school, 4 = college, 5 = University of applied sciences, 6 = University); Age cohort (1 = 18–34, 2 = 35–49, 3 = 50–64, 4 = 65–89); Study sample (1 = National sample, 2 = PSHVA, sample). Numbers in parentheses indicate non-invariant parameters for that item-group combination. Loading non-invariance percentage represents the proportion of factor loading parameters that differ significantly across groups within each demographic variable. Intercepts non-invariance percentage represents the proportion of intercept parameters that differ significantly across groups. Total non-invariance percentage indicates the overall proportion of measurement parameters (both loadings and intercepts) showing group differences.Alignment optimization employed fixed estimation with reference group constraints. Acceptable threshold for group comparisons: ≤25% non-invariant parameters.

### Latent Mean Comparisons

The results of MC simulations with 1,000 replications for two different sample sizes (see details in Supplementary Table S4) confirmed reliable parameter recovery across all demographic comparisons (r = 0.87–1.00) [27]. Stratified analyses showed consistent patterns across national and regional samples, supporting result robustness. Group comparisons are therefore reported in Table 5 using the combined sample for optimal statistical precision (details in Supplementary Table S5).

**TABLE 5:** Comparisons of latent factor means of HLS-Q12 across sociodemographic groups (Health and wellbeing survey, Finland, 2024).

Category	Groups	Factor mean
Gender	Male (reference)	
​	Female	0.388*
Age cohort	18–34 (reference)	—
​	35–49	−0.057
​	50–64	−0.015
​	65–89	−0.124*
Education	Basic education (reference)	​
​	Vocational upper secondary education	0.188*
​	General upper secondary education	0.198*
​	Post-secondary non-tertiary education	0.316*
​	University of applied sciences	0.516*
​	University	0.556*
Study sample	Regional sample (reference)	—
​	National sample	−0.233*

Latent factor means were estimated using Mplus Alignment Optimization with ALIGNMENT = FIXED (reference group’s mean = 0, variance = 1). Asterisks (*) indicate significant pairwise Wald z contrasts vs. the reference group, (two-sided α = .05; SEs of robust maximum likelihood estimation). Latent means are not observed means. Demographic patterns were consistent with slight variation across national and regional subsamples. Details in Supplementary Table S5.

Significant demographic differences emerged across all examined variables. Women scored higher than men (0.388), with clear educational gradients favoring higher education levels. Younger adults (18–34) exhibited higher estimated latent means than older adults (65+: -0.124), while middle-aged groups showed intermediate levels. The regional sample scored higher than the national sample (0.233).

## Discussion

This study applied complementary analytic approaches to examine the internal structure and cross-group comparability of the HLS-Q12 among Finnish adults. By combining traditional MGCFA with alignment optimization, we provide evidence for largely adequate measurement invariance across key sociodemographic groups and summarize subgroup patterns relevant for population monitoring in Finland.

The HLS-Q12 exhibited robust psychometric properties in the Finnish context, with excellent internal consistency and a clear unidimensional factor structure. These findings are broadly consistent with previous studies in other settings [16–18], supporting the feasibility of the HLS-Q12 across diverse populations. CFA results supported the theoretical framework underlying health literacy as a unified construct encompassing competencies across healthcare, disease prevention, and health promotion domains [1].

Our sequential analytical approach demonstrated the complementary value of traditional and innovative methods for testing measurement invariance. The alignment optimization method provided more nuanced insights than conventional MGCFA by identifying specific non-invariant parameters while accommodating partial invariance conditions. The Monte Carlo validation feature offered empirical assessment of parameter reliability unavailable in traditional approaches, with most demographic comparisons achieving excellent recovery. This methodological framework provides a replicable template for cross-cultural validation studies.

The demographic patterns observed provide important insights for Finnish health and social policy as well as international health literacy research. Higher health literacy scores among women are consistent with prior European evidence [11, 13, 39], likely reflecting greater healthcare engagement and health information-seeking behaviors. The educational gradient demonstrated clear benefits of education at all levels, with university graduates showing the highest health literacy and those with only basic education showing the largest deficits. This pattern reflects the well-documented association between educational attainment and health literacy capabilities [4, 40], highlighting the importance of educational equity for population health literacy in Finland.

The age-related patterns revealed young adults demonstrating the highest health literacy levels, showing higher estimated latent means than older adults, consistent with previous studies [41] and recent observations in Germany [42]. However, comparisons among younger and middle-aged groups showed limited significant differences, suggesting that major health literacy decline primarily occurs after age 65 rather than following a linear age gradient [43]. The relative advantage of younger adults, though not always statistically significant compared to middle-aged groups, may reflect their greater familiarity with digital health information environments that increasingly characterize modern healthcare systems [44]. This interpretation gains particular relevance in the Nordic context, where digital health services are rapidly expanding and require advanced information processing skills beyond traditional health literacy competencies, and require further studies to explore the mechanisms underlying generational differences in health literacy acquisition and expression [45].

Moreover, the finding that the regional sample demonstrated slightly higher health literacy than the national sample was also unexpected, given that rural and regional populations typically show lower health literacy in international studies [13, 46, 47]. However, place of residence, whether rural or regional, is not necessarily an independent determinant of health literacy [46]. Regional variations in this study may reflect either genuine contextual factors or methodological influences such as sampling differences, warranting cautious interpretation and further investigation with standardized recruitment approaches.

This study has several notable strengths. First, the large sample size with broad sociodemographic coverage provides robust statistical power for subgroup analyses. Second, the inclusion of both a national sample and a regional North Savo sample enables examination of sampling source effects and strengthens the robustness of subgroup comparisons. Third, the combined use of traditional and innovative method offers complementary evidence for cross-group comparability under realistic conditions [48–51]. Fourth, Monte Carlo simulations provided additional support for the stability of the main estimation results.

However, certain methodological considerations should be acknowledged. First, this study provides evidence primarily for internal structure, internal consistency, and measurement invariance across key sociodemographic groups. We did not examine other sources of validity evidence, including convergent and discriminant validity with alternative health literacy instruments, criterion validity against external benchmarks, or predictive validity for subsequent outcomes. Although subgroup differences are reported after establishing measurement invariance, these comparisons are intended for population monitoring and should not be interpreted as formal known-groups validity evidence in the absence of external criteria. Future research should strengthen the validity evidence base by linking the HLS-Q12 to theoretically relevant external criteria and longitudinal outcomes.

Second, the Finnish version was developed and reviewed within the research team and did not include a formal forward-backward translation protocol with systematic cognitive debriefing, which may affect cross-cultural comparability. Future work can strengthen semantic and conceptual equivalence by applying standardized translation and cultural adaptation procedures, including cognitive interviewing.

Third, the cross-sectional design limits temporal inference. The sampling approach, which combined a nationally representative online panel with a regional sample using multiple recruitment channels, may affect generalizability. Potential selection bias should be considered when interpreting subgroup differences, particularly for national versus regional comparisons. In addition, health literacy was measured using self-reported perceived difficulty items, which may be influenced by response styles.

These findings support the feasibility of monitoring population health literacy using an internationally recognized instrument. Our findings demonstrate that health literacy varies significantly across demographic groups in predictable patterns, with education and age emerging as key correlates requiring targeted public health attention. The successful application of both traditional and innovative measurement invariance methods confirms that meaningful group comparisons are possible despite minor measurement variations, providing confidence for health policy applications. Most importantly, this study enables Finland to contribute to and benefit from international health literacy research [52], supporting evidence-based strategies and cross-national learning essential to address health information challenges in increasingly complex health context. Future research should link HLS-Q12 scores to external criteria and longitudinal outcomes, and evaluate responsiveness in intervention and policy contexts.

## References

[1] Sørensen K Van den Broucke S Fullam J Doyle G Pelikan J Slonska Z Health Literacy and Public Health: A Systematic Review and Integration of Definitions and Models. BMC Public Health (2012) 12(1):80. 10.1186/1471-2458-12-80 22276600 PMC3292515

[2] Smith C Behan S Belton S Nicholl C Murray M Goss H . An Update on Health Literacy Dimensions: An Umbrella Review. PLOS ONE (2025) 20(6):e0321227. 10.1371/journal.pone.0321227 40493611 PMC12151441

[3] WHO. Health Literacy. Geneva, Switzerland: World Health Organization (2025). Available online at: https://www.who.int/news-room/fact-sheets/detail/health-literacy (Accessed January 22, 2026).

[4] Berkman ND Sheridan SL Donahue KE Halpern DJ Crotty K . Low Health Literacy and Health Outcomes: An Updated Systematic Review. Ann Intern Med (2011) 155(2):97–107. 10.7326/0003-4819-155-2-201107190-00005 21768583

[5] Suen K Shrestha S Osman S Paudyal V . Association Between Patient Race/Ethnicity, Health Literacy, Socio-Economic Status, and Incidence of Medication Errors: A Systematic Review. J Racial Ethn Health Disparities (2025). 10.1007/s40615-025-02407-8 40180697 PMC13157403

[6] Fan Zya Yang Y Zhang F . Association Between Health Literacy and Mortality: A Systematic Review and Meta-Analysis. Arch Public Health (2021) 79(1):119. 10.1186/s13690-021-00648-7 34210353 PMC8247180

[7] Shahid R Shoker M Chu LM Frehlick R Ward H Pahwa P . Impact of Low Health Literacy on Patients’ Health Outcomes: A Multicenter Cohort Study. BMC Health Serv Res (2022) 22(1):1148. 10.1186/s12913-022-08527-9 36096793 PMC9465902

[8] de Buhr E Tannen A . Parental Health Literacy and Health Knowledge, Behaviours and Outcomes in Children: A Cross-Sectional Survey. BMC Public Health (2020) 20(1):1096. 10.1186/s12889-020-08881-5 32660459 PMC7359277

[9] Lee HY Zhou AQ Lee RM Dillon AL . Parents’ Functional Health Literacy Is Associated with Children’s Health Outcomes: Implications for Health Practice, Policy, and Research. Child Youth Serv Rev (2020) 110:104801. 10.1016/j.childyouth.2020.104801

[10] Ishizumi A Kolis J Abad N Prybylski D Brookmeyer KA Voegeli C Beyond Misinformation: Developing a Public Health Prevention Framework for Managing Information Ecosystems. Lancet Public Health (2024) 9(6):e397–406. 10.1016/S2468-2667(24)00031-8 38648815 PMC11369959

[11] Paakkari L Torppa M Mazur J Boberova Z Sudeck G Kalman M A Comparative Study on Adolescents’ Health Literacy in Europe: Findings from the HBSC Study. Int J Environ Res Public Health (2020) 17(10):3543. 10.3390/ijerph17103543 32438595 PMC7277198

[12] Seng JJB Yeam CT Huang CW Tan NC Low LL . Pandemic-Related Health Literacy: A Systematic Review of Literature in COVID-19, SARS and MERS Pandemics. Singapore Med J (2023) 66(5):244–55. 10.4103/singaporemedj.SMJ-2021-026 37459004 PMC12161643

[13] Sørensen K Pelikan JM Röthlin F Ganahl K Slonska Z Doyle G Health Literacy in Europe: Comparative Results of the European Health Literacy Survey (HLS-EU). Eur J Public Health (2015) 25(6):1053–8. 10.1093/eurpub/ckv043 25843827 PMC4668324

[14] Kiechle ES Bailey SC Hedlund LA Viera AJ Sheridan SL . Different Measures, Different Outcomes? A Systematic Review of Performance-Based Versus Self-Reported Measures of Health Literacy and Numeracy. J Gen Intern Med (2015) 30(10):1538–46. 10.1007/s11606-015-3288-4 25917656 PMC4579206

[15] Storms H Claes N Aertgeerts B Van den Broucke S . Measuring Health Literacy Among Low Literate People: An Exploratory Feasibility Study with the HLS-EU Questionnaire. BMC Public Health (2017) 17(1):475. 10.1186/s12889-017-4391-8 28526009 PMC5438531

[16] Finbråten HS Wilde-Larsson B Nordström G Pettersen KS Trollvik A Guttersrud Ø . Establishing the HLS-Q12 Short Version of the European Health Literacy Survey Questionnaire: Latent Trait Analyses Applying Rasch Modelling and Confirmatory Factor Analysis. BMC Health Serv Res (2018) 18(1):506. 10.1186/s12913-018-3275-7 29954382 PMC6022487

[17] Zanini DS Peixoto EM de Andrade JM Fernandes IA da Silva MPP . European Health Literacy Survey Questionnaire Short Form (HLS-Q12): Adaptation and Evidence of Validity for the Brazilian Context. Psicol Reflex E Crítica (2023) 36(1):25. 10.1186/s41155-023-00263-1 37672100 PMC10482809

[18] Islam FMA . Study of the Psychometric Properties of the HLS-EU-12 Questionnaire in Rural Bangladesh. PLOS ONE (2025) 20(5):e0323608. 10.1371/journal.pone.0323608 40354386 PMC12068572

[19] Sørensen K Van den Broucke S Pelikan JM Fullam J Doyle G Slonska Z Measuring Health Literacy in Populations: Illuminating the Design and Development Process of the European Health Literacy Survey Questionnaire (HLS-EU-Q). BMC Public Health (2013) 13(1):948. 10.1186/1471-2458-13-948 24112855 PMC4016258

[20] Putnick DL Bornstein MH . Measurement Invariance Conventions and Reporting: The State of the Art and Future Directions for Psychological Research. Dev Rev (2016) 41:71–90. 10.1016/j.dr.2016.06.004 27942093 PMC5145197

[21] Ilmarinen KM Aalto AM Muuri AL . Unmet Need for and Barriers to Receiving Health Care and Social Welfare Services in Finland. Scand J Public Health (2025) 53(1 Suppl. l):52–63. 10.1177/14034948241299019 39663697 PMC11951374

[22] Laukka E Lakoma S Harjumaa M Hiltunen S Härkönen H Jansson M Older Adults’ Preferences in the Utilization of Digital Health and Social Services: A Qualitative Analysis of Responses to Open-Ended Questions. BMC Health Serv Res (2024) 24(1):1184. 10.1186/s12913-024-11564-1 39367429 PMC11451244

[23] Eronen J Paakkari L Portegijs E Saajanaho M Rantanen T . Health Literacy Supports Active Aging. Prev Med (2021) 143:106330. 10.1016/j.ypmed.2020.106330 33220399

[24] Summanen AM Rautopuro J Kannas L Paakkari L . Measuring Health Literacy in Basic Education in Finland: The Development of a Curriculum- and Performance-Based Measurement Instrument. Int J Environ Res Public Health (2022) 19(22):15170. 10.3390/ijerph192215170 36429888 PMC9690733

[25] Leitgöb H Seddig D Asparouhov T Behr D Davidov E De Roover K Measurement Invariance in the Social Sciences: Historical Development, Methodological Challenges, State of the Art, and Future Perspectives. Soc Sci Res (2023) 110:102805. 10.1016/j.ssresearch.2022.102805 36796989

[26] Chen FF . Sensitivity of Goodness of Fit Indexes to Lack of Measurement Invariance. Struct Equ Model Multidiscip J (2007) 14(3):464–504. 10.1080/10705510701301834

[27] Asparouhov T Muthén B . Multiple-Group Factor Analysis Alignment. Struct Equ Model Multidiscip J (2014) 21(4):495–508. 10.1080/10705511.2014.919210

[28] Muthén B Asparouhov T . Recent Methods for the Study of Measurement Invariance with Many Groups: Alignment and Random Effects. Sociol Methods Res (2018) 47(4):637–64. 10.1177/0049124117701488

[29] Flake JK McCoach DB . An Investigation of the Alignment Method with Polytomous Indicators Under Conditions of Partial Measurement Invariance. Struct Equ Model Multidiscip J (2018) 25(1):56–70. 10.1080/10705511.2017.1374187

[30] Marsh HW Guo J Parker PD Nagengast B Asparouhov T Muthén B What to Do when Scalar Invariance Fails: The Extended Alignment Method for Multi-Group Factor Analysis Comparison of Latent Means Across Many Groups. Psychol Methods (2018) 23(3):524–45. 10.1037/met0000113 28080078

[31] TENK. Guidelines for Ethical Review in Human Sciences (2019). Available online at: https://tenk.fi/en/advice-and-materials/guidelines-ethical-review-human-sciences (Accessed January 23, 2026).

[32] IBM Corp. IBM SPSS Statistics for Windows, Version 29.0. Armonk, NY (2022).

[33] Muthén LK Muthén BO . Mplus User’s Guide. 8th ed. Los Angeles, CA: Muthén & Muthén (2017). Available online at: https://www.statmodel.com/html_ug.shtml (Accessed March 4, 2025).

[34] Yuan KH Bentler PM . Normal Theory Based Test Statistics in Structural Equation Modelling. Br J Math Stat Psychol (1998) 51(2):289–309. 10.1111/j.2044-8317.1998.tb00682.x 9854947

[35] Hu L Bentler PM . Cutoff Criteria for Fit Indexes in Covariance Structure Analysis: Conventional Criteria Versus New Alternatives. Struct Equ Model Multidiscip J (1999) 6(1):1–55. 10.1080/10705519909540118

[36] Kline RB . Principles and Practice of Structural Equation Modeling. New York, NY: Guilford Publications (2023). p. 514.

[37] Byrne B Van De Vijver F . The Maximum Likelihood Alignment Approach to Testing for Approximate Measurement Invariance: A Paradigmatic Cross-Cultural Application. Psicothema (2017) 4(29):539–51. 10.7334/psicothema2017.178 29048316

[38] Brown TA . Confirmatory Factor Analysis for Applied Research. 2nd ed. New York, NY: Guilford Publications (2015). p. 482.

[39] Pelikan JM Link T Straßmayr C Waldherr K Alfers T Bøggild H Measuring Comprehensive, General Health Literacy in the General Adult Population: The Development and Validation of the HLS19-Q12 Instrument in Seventeen Countries. Int J Environ Res Public Health (2022) 19(21):14129. 10.3390/ijerph192114129 36361025 PMC9659295

[40] Stormacq C Van den Broucke S Wosinski J . Does Health Literacy Mediate the Relationship Between Socioeconomic Status and Health Disparities? Integrative Review. Health Promot Int (2019) 34(5):e1–17. 10.1093/heapro/day062 30107564

[41] Kobayashi LC Wardle J Wolf MS von Wagner C . Aging and Functional Health Literacy: A Systematic Review and Meta-Analysis. J Gerontol Ser B (2016) 71(3):445–57. 10.1093/geronb/gbu161 25504637 PMC4834761

[42] Schaeffer D Berens EM Gille S Griese L Klinger J de Sombre S Gesundheitskompetenz Der Bevölkerung in Deutschland Vor Und Während Der Corona Pandemie: Ergebnisse Des HLS-GER 2. Universität Bielefeld, Interdisziplinäres Zentrum Für Gesundheitskompetenzforschung (2021). Available online at: https://pub.uni-bielefeld.de/record/2950305 (Accessed September 25, 2025).

[43] Klinger J Berens EM Schaeffer D . Health Literacy and the Role of Social Support in Different Age Groups: Results of a German Cross-Sectional Survey. BMC Public Health (2023) 23(1):2259. 10.1186/s12889-023-17145-x 37974154 PMC10652531

[44] Norman CD Skinner HA . Ehealth Literacy: Essential Skills for Consumer Health in a Networked World. J Med Internet Res (2006) 8(2):e506. 10.2196/jmir.8.2.e9 16867972 PMC1550701

[45] Griebel L Enwald H Gilstad H Pohl AL Moreland J Sedlmayr M . Ehealth Literacy research—Quo Vadis? Inform Health Soc Care (2018) 43(4):427–42. 10.1080/17538157.2017.1364247 29045164

[46] Aljassim N Ostini R . Health Literacy in Rural and Urban Populations: A Systematic Review. Patient Educ Couns (2020) 103(10):2142–54. 10.1016/j.pec.2020.06.007 32601042

[47] Batterham RW Hawkins M Collins PA Buchbinder R Osborne RH . Health Literacy: Applying Current Concepts to Improve Health Services and Reduce Health Inequalities. Public Health (2016) 132:3–12. 10.1016/j.puhe.2016.01.001 26872738

[48] Ding Y Yang Hansen K Klapp A . Testing Measurement Invariance of Mathematics Self-Concept and Self-Efficacy in PISA Using MGCFA and the Alignment Method. Eur J Psychol Educ (2023) 38(2):709–32. 10.1007/s10212-022-00623-y

[49] Luong R Flake JK . Measurement Invariance Testing Using Confirmatory Factor Analysis and Alignment Optimization: A Tutorial for Transparent Analysis Planning and Reporting. Psychol Methods (2023) 28(4):905–24. 10.1037/met0000441 35588078

[50] Rudnev M . Alignment Method for Measurement Invariance: Tutorial. Elem Cross-Cultural Research (2019). Available online at: https://maksimrudnev.com/2019/05/01/alignment-tutorial/(Accessed August 7, 2025).

[51] Sözer BE . Evaluating Measurement Invariance of Students’ Practices Regarding Online Information Questionnaire in PISA 2022: A Comparative Study Using MGCFA and Alignment Method. Educ Inf Technol (2025) 30(1):1219–37. 10.1007/s10639-024-12921-7

[52] Dietscher C Pelikan J . The Action Network for Measuring Population and Organizational Health Literacy (M-POHL) and Its Health Literacy Survey 2019 (HLS19). Eur J Public Health (2019) 29(Suppl. ment_4):ckz185.556. 10.1093/eurpub/ckz185.556

