# Supplementary material for: Structural Validation and Measurement Invariance of the HLS-Q12 Health Literacy Instrument in Finnish Adults: Comparing Traditional and Alignment Methods
Source: Int J Public Health. 2026 Mar 26;71:1609337. doi: 10.3389/ijph.2026.1609337 (PMC13061856; doi:10.3389/ijph.2026.1609337)
Supplement: Supplementary file 1 [file DataSheet1.pdf]

Supplementary Table 1 Descriptive statistics for HLS-Q12 items

Items	N	Mean	SD	Skewness	Kurtosis
1. Find information on treatment of illnesses you have?	7,054	3.25	0.67	-0.55	0.13
2. Understand what to do in medical emergencies?	7,057	3.26	0.66	-0.53	0.09
3. Judge the advantages and disadvantages of different treatment options?	7,054	2.86	0.75	-0.15	-0.43
4. Follow the instructions on medication?	7,043	3.53	0.59	-1.00	0.84
5. Find information on how to manage mental health problems like stress and depression?	7,037	3.09	0.74	-0.51	0.00
6. Understand why you need health screenings?	7,040	3.38	0.67	-0.85	0.56
7. Judge if the information on health risks in the media is reliable?	7,047	2.95	0.76	-0.34	-0.23
8. Decide how you can protect yourself from illnesses based on advice from family and friends?	7,032	3.05	0.69	-0.36	0.06
9. Find information on health attitudes such as exercise, healthy food and nutrition?	7,033	3.37	0.64	-0.68	0.32
10. Understand information on food packaging?	7,031	3.06	0.76	-0.45	-0.27
11. Judge which everyday behaviors are related to health?	7,022	3.29	0.63	-0.50	0.13
12. Make decisions to improve your health?	6,974	2.96	0.78	-0.35	-0.37

Note. Scale range: 1–4; higher scores indicate higher perceived health literacy. SD = Std. Deviation

Supplementary Table 2 Approximate measurement invariance for intercepts and factor loadings of items (alignment optimization) across groups in national sample

	**Loading invariance**											
Item number	Gender		Education						Age cohort			
Q1	1	2	1	2	3	4	5	6	1	2	3	4
Q2	1	2	1	2	3	4	5	6	1	2	3	4
Q3	1	2	1	2	3	4	5	6	1	2	3	4
Q4	1	2	1	2	3	4	5	6	1	2	3	4
Q5	1	2	1	2	3	4	5	6	1	2	3	4
Q6	1	2	1	2	3	4	5	6	1	2	3	4
Q7	1	2	1	2	3	4	5	6	1	2	3	4
Q8	1	2	1	2	3	4	5	6	1	2	3	4
Q9	1	2	1	2	3	4	5	6	1	2	3	4
Q10	1	2	1	2	3	4	5	6	1	2	3	4
Q11	1	2	1	2	3	4	5	6	1	2	3	4
Q12	1	2	1	2	3	4	5	6	1	2	3	4
Loadings noninvariance %	0.0%		0.0%						0.0%			
	**Intercepts invariance**											
Item number	Gender		Education						Age cohort			
Q1	1	2	1	2	3	4	5	6	1	2	3	4
Q2	1	2	1	(2)	3	4	5	6	1	2	3	4
Q3	(1)	(2)	(1)	(2)	3	4	5	6	(1)	2	3	4
Q4	1	2	1	2	3	4	5	6	(1)	2	3	(4)
Q5	1	2	1	2	3	4	5	6	1	2	3	4
Q6	(1)	(2)	1	2	3	4	5	6	(1)	(2)	3	4
Q7	1	2	1	2	3	4	5	6	(1)	2	3	(4)
Q8	(1)	(2)	1	2	3	4	5	6	1	2	3	4
Q9	(1)	(2)	1	2	3	4	5	6	(1)	2	3	4
Q10	(1)	(2)	1	2	3	4	5	6	1	2	(3)	4
Q11	1	2	1	2	3	4	5	6	1	2	3	4
Q12	(1)	(2)	1	2	3	4	5	6	1	2	3	(4)
Intercepts noninvariance %	50.0%		4.2%						20.8%			

Total noninvariance %	25.0%	2.1%	10.4%

Numbers represent group classifications: Gender (1 = male, 2 = female); Education (1 = basic education, 2 = Vocational upper secondary education, 3 = , General upper secondary education, 4 = Post-secondary non-tertiary education, 5 = University of applied sciences, 6 = University); Age cohort (1 = 18-34, 2 = 35-49, 3 = 50-64, 4 = 65-89).
Numbers in parentheses indicate non-invariant parameters for that item-group combination.
Loading non-invariance percentage represents the proportion of factor loading parameters that differ significantly across groups within each demographic variable.
Intercepts non-invariance percentage represents the proportion of intercept parameters that differ significantly across groups.
Total non-invariance percentage indicates the overall proportion of measurement parameters (both loadings and intercepts) showing group differences.
Alignment optimization employed fixed estimation with reference group constraints.
Acceptable threshold for group comparisons: ≤25% non-invariant parameters.

Supplementary Table 3 Approximate measurement invariance for intercepts and factor loadings of items (alignment optimization) across groups in North Savo sample

	**Loading invariance**											
Item number	Gender		Education						Age cohort			
Q1	1	2	1	2	3	4	5	6	1	2	3	4
Q2	1	2	1	2	3	4	5	6	1	2	3	4
Q3	1	2	1	2	3	4	5	6	1	2	3	4
Q4	1	2	1	2	3	4	5	6	1	2	3	4
Q5	1	2	1	2	3	4	5	6	1	2	3	4
Q6	1	2	1	2	3	4	5	6	1	2	3	4
Q7	1	2	1	2	3	4	5	6	1	2	3	4
Q8	1	2	1	2	3	4	5	6	1	2	3	4
Q9	1	2	1	(2)	3	4	5	6	1	2	3	4
Q10	1	2	1	2	3	4	5	6	1	2	3	4
Q11	1	2	1	2	3	4	5	6	1	2	3	4
Q12	1	2	1	2	3	4	5	6	1	2	3	4
Loadings noninvariance %	0.0%		1.4%						0.0%			
	**Intercepts invariance**											
Item number	Gender		Education						Age cohort			
Q1	1	2	(1)	(2)	3	4	5	6	1	2	3	(4)
Q2	1	2	1	2	3	4	5	6	1	2	3	(4)
Q3	(1)	(2)	1	2	3	4	5	6	1	2	3	(4)
Q4	1	2	1	2	3	4	5	6	1	2	3	4
Q5	1	2	1	2	3	4	5	6	1	2	3	4
Q6	1	2	1	2	3	4	5	6	1	2	(3)	(4)
Q7	1	2	1	2	3	4	5	6	1	2	3	(4)
Q8	1	2	1	2	3	4	5	6	1	2	3	4
Q9	1	2	1	2	3	4	5	6	(1)	2	3	4
Q10	1	2	1	2	3	4	5	6	1	2	3	4
Q11	1	2	1	2	3	4	5	6	1	2	3	4
Q12	(1)	(2)	1	2	3	4	5	6	1	2	3	(4)
Intercepts noninvariance %	16.7%		2.8%						16.7%			

Total noninvariance %	8.3%	2.1%	8.3%

Numbers represent group classifications: Gender (1 = male, 2 = female); Education (1 = basic education, 2 = Vocational upper secondary education, 3 = , General upper secondary education, 4 = Post-secondary non-tertiary education, 5 = University of applied sciences, 6 = University); Age cohort (1 = 18-34, 2 = 35-49, 3 = 50-64, 4 = 65-89).
Numbers in parentheses indicate non-invariant parameters for that item-group combination.
Loading non-invariance percentage represents the proportion of factor loading parameters that differ significantly across groups within each demographic variable.
Intercepts non-invariance percentage represents the proportion of intercept parameters that differ significantly across groups.
Total non-invariance percentage indicates the overall proportion of measurement parameters (both loadings and intercepts) showing group differences.
Alignment optimization employed fixed estimation with reference group constraints.
Acceptable threshold for group comparisons: ≤25% non-invariant parameters.

Supplementary Table 4 Results of Monte Carlo simulation (Correlations of Population and Estimate Values) for HLS-Q12

	**Sample size**	**nrep: 1,000**	**Gender**		**Education**		**Age cohort**		**Data source**	
		Estimated statistics	Average	SD	Average	SD	Average	SD	Average	SD
Full sample	original	Factor mean	1.0000	0.0000	0.9911	0.0067	0.9200	0.0905	1.0000	0.0000
		Factor variance	0.9960	0.0894	0.8813	0.1432	0.2326	0.5609	0.9880	0.1545
	5,000	Factor mean	1.0000	0.0000	0.9981	0.0013	0.9722	0.2860	1.0000	0.0000
		Factor variance	1.0000	0.0000	0.9787	0.0152	0.3443	0.5255	1.0000	0.0000
National sample	original	Factor mean	1.0000	0.0000	0.9714	0.0234	0.8675	0.1533	/	/
		Factor variance	0.9580	0.2869	0.8310	0.1713	0.6725	0.3483	/	/
	5,000	Factor mean	1.0000	0.0000	0.9969	0.0022	0.9716	0.0298	/	/
		Factor variance	1.0000	0.0000	0.9813	0.0133	0.9295	0.0766	/	/
North Savo sample	original	Factor mean	1.0000	0.0000	0.9845	0.0117	0.8995	0.1190	/	/
		Factor variance	1.0000	0.0000	0.8598	0.1463	0.7436	0.2674	/	/
	5,000	Factor mean	1.0000	0.0000	0.9964	0.0019	0.9826	0.0180	/	/
		Factor variance	1.0000	0.0000	0.9883	0.0084	0.9608	0.0402	/	/

nrep = number of replications; SD = standard deviation

Supplementary Table 5 Comparisons of factor means of HLS-Q12 across diverse groups

			Total sample		National Sample		North Savo sample	
Category	Groups IDs		Factor mean	Sig. pattern	Factor mean	Sig. pattern	Factor mean	Sig. pattern
**Gender**	**1**	**Male (reference)**						
	2	Female	0.388	> group 1 *	0.317	> male *	0.353	> male *
**Age**	**1**	**18-34 (reference)**						
	2	35-49	-0.057	> group 4 *	-0.104	< group 1*	0.018	> group 4 *
	3	50-64	-0.015		-0.034		0.000	> group 4 *
	4	65-89	-0.124	< group 1, 2*	-0.114	< group 1*	-0.129	< group 1*
**Education**	**1**	**Basic education (reference)**						
	2	Vocational upper secondary education	0.188	> group 1*	0.144	> group 1*	0.196	> group 1*
	3	General upper secondary education	0.198	> group 1*	0.207	> group 1*	0.149	
	4	Post-secondary non-tertiary education	0.316	> group 3, 2, 1*	0.301	> group 2, 1*	0.270	> group 1*
	5	University of applied sciences	0.516	> group 4, 3, 2, 1*	0.426	> group 4, 3, 2, 1*	0.561	> group 4, 3, 2, 1*
	6	University	0.556	> group 4, 3, 2, 1*	0.394	> group 3, 2, 1*	0.692	> group 5, 4, 3, 2, 1*
**Stuy sample**	**1**	**Regional sample (reference)**			/	/	/	/
	2	National sample	-0.233	< group 1 *	/	/	/	/

Latent factor means were estimated using Mplus Alignment Optimization with ALIGNMENT=FIXED (reference group's mean = 0, variance = 1).
Asterisks (*) indicate denote pairwise Wald z tests (two-sided α=.05; robust MLR SEs).
No multiple-comparison adjustment.
The column "Sig. pattern" indicate significant pairwise differences (row vs comparator) listing groups for which the row group has a significantly higher latent mean (symbol ">") or lower latent mean (symbol "<") in pairwise Wald z tests.
Entries are latent factor means on the latent scale and are not observed means.
Each panel (Total, National, North Savo) comes from a separate alignment run; latent scales are panel-specific and not directly comparable across panels.
